# A Small Independent Inquiry Commission Called for

**Published:** 1919-09-20

**Authors:** 


### A Small Independent Inquiry Commission Called For.

The present position of affairs in this hospital as recorded to us, and the letter of the Secretary we publish above, demonstrate the paramount necessity there is for the appointment of a small independent, authoritative commission to investigate the actual state of affairs at Queen Charlotte's Hospital as it exists throughout. This hospital was founded in 1752, and, though incorporated in 1885, there is evidence to fear that the old traditions and conditions have found a permanent place within its walls. "The Committee hope for some diminution in the hours of duty of pupils." As they stand at present it causes wonder that women can be found in considerable numbers to pay £40 for six months' training, submit to the conditions reported, and board and lodging. We have before us an example of the dietary supplied to the night duty staff on Wednesday, September 10. "Breakfast 7.30 p.m., haddock, from which the freshness had disappeared some time previously; it was left uneaten; tea and bread and butter. Four and a half hours later, *i.e.*, at midnight, pieces of bread and cheese with coffee. Four and a half hours later, *i.e.*, at 4.30 a.m., a piece of bread and dripping. Some five hours later, *i.e.*, at 9.20 a.m., roast mutton and vegetables, the mutton so badly cooked that much of it was wasted." It is a pleasant irony, we understand, to speak of cooking where the food supplied to the pupils of this hospital is concerned. This is not the worst type of domestic service imposed on the night staff. An unfortunate pupil nurse has to prepare these meals and wash up, in addition to attending to the needs of three lying-in mothers and their babies.

The condition of single bedrooms may be judged—(*a*) "to open a window black filth comes on to the hands"; (*b*) the old converted houses have living creatures known as "Norfolk Howards" in their crevices; samples of the live stock have been supplied to the night sister. The service and crockery are shameless. The Secretary admits the day nurses' hours are fourteen, with two hours off duty each day, subject to fluctuations we understand. This hospital has the unenviable notoriety of providing that every nurse within its walls shall pay to the hospital heavy fees or give free service for a whole year. The account of the cleaning of the wards and the rough work imposed on the nurses given by the Secretary is not inviting if it is even justifiable. If an ample supply of inviting. if it is even justifiable. If an ample supply of We have evidence that cleaning materials, if supplied by the hospital, do not reach the pupil nurses. Indeed, a request from a new pupil to the staff nurse for brass polish received the reply that she must supply her own. It would appear therefore that the authorities are unaware of the internal working and practices within their institution. For "the pupils, it is avowed, have to purchase such commodities with their own money and the practice is well-known and countenanced."

Another remarkable practice is that the pupil nurse who has had the courage to stand ten days of these recorded conditions can escape by resignation aften ten days' service and take with her £30 of the full fee, the hospital retaining £10 of the pupil's small savings. The mystery is why the whole of the pupil nurses do not go to the hospital office, draw their £30, and depart in a body. What are the leaders of the Labour Party about that they allow such treatment of women workers to continue without protest or redress? Two representatives of the Labour Party, one a man and one a woman, might properly form members of a small commission of five charged with the duty of thoroughly investigating the conditions which have and do prevail at Queen Charlotte's Hospital. The institution would seem to be conducted on the principle that what has been good enough in the long past is good enough now. Have the administration yet heard of The War or the League of Nations, or better days for working men and women, to say nothing of *working midwives and those who work them presumably at a profit*?

There remains off-duty time. At this hospital it is stated that "two days off a month sound well," but it has proved in practice a continual uncertainty. Sometimes two and even three of the off days have been entirely lost by the nurses "owing to circumstances intervening." The two hours daily are declared to be often withheld, curtailed or reduced, sometimes "owing to circumstances or the whims of the immediate superior in rank."

It is certified that the absence of better working conditions and facilities of study and attendance at class lectures is gravely compromising to the conductors of this hospital and those responsible for its affairs. It is our clear duty to ask Viscount Portman as the President, Sir Samuel Scott, Bart., M.P., Chairman, with the responsible managers, to justify the continual treatment of ninety cases a day within this hospital when it is only equipped and staffed for sixty. That is a grave question affecting the interest and welfare of large numbers of poor people, mothers and children, and we must press for an immediate answer. The best managed lying-in hospitals properly publish the sums received from nurses and probationers' fees. Queen Charlotte's Hospital avoids taking this proper course, and it is a grave omission.

The Ed. of The Hospital.

